# The evaluation of parameter effects on cefoperazone treatability with new generation anodes

**DOI:** 10.1038/s41598-022-18486-0

**Published:** 2022-08-18

**Authors:** Ayşe Kurt, Taner Yonar

**Affiliations:** 1 Central Research Laboratory, Bursa Uludag University, Görükle Campus, 16059 Bursa, Turkey; 2 Environmental Engineering, Faculty of Engineering, Bursa Uludag University, Görükle Campus, 16059 Bursa, Turkey

**Keywords:** Environmental chemistry, Environmental impact, Environmental sciences, Chemistry

## Abstract

In this study it was aimed to investigate the treatability of cefoperazone with new generation Sb-doped SnO_2_-Ni anodes. For this purpose, it was studied with Sn/Sb/Ni: 500/8/1 anodes for the oxidation of aqueous solution containing cefoperazone antibiotic by addition of different types of electrolyte. Potassium chloride was found as the best electrolyte type affecting the electrochemical reactions positively even at lower concentrations (750 mg/L^−1^). At pH 8 the best results were obtained, which is the neutral pH value of the aqueous solution. 50 mA/cm^2^ was found as the best value for current density parameter, providing full mineralization just after 60 min of reaction. The removal efficiencies increased generally with the increase of current density, because active oxidants occur increasingly at higher current values. According to the results of the study it was seen that, electrochemical oxidation processes with Sn/Sb/Ni–Ti anodes could be carried out efficiently without need adding extra electrolyte (salt) and pH adjustment step for real wastewaters containing antibiotics. Thus, it was found an easy and economic way to perform electrochemical oxidation with Sn/Sb/Ni–Ti anodes for the wastewaters containing cefoperazone antibiotics.

## Introduction

Antibiotics are used for many years to treat diseases related to bacterial infections for human and animal health, for fish farm practices, and support the growth of animals^1,2^. However, they cause to the adverse effects on the environment directly or indirectly through contamination and consequently, they may cause to bacterial resistance in the environment at long term. Thus, they could be considered as the most dangerous pollutant types due to have toxicologic properties causing to the microorganism resistance in the environment^3,4^. Bacteria with antibiotic resistance genes can spread easily in the environment, threatening human health potentially^5^. However, they are also known for having endocrine disrupting properties.

Endocrine disrupting compounds (EDCs) are known to disrupt endocrine systems in the human body. The presence of these compounds in the environment causes great deterioration in human and environmental health^6^. Recent studies show that, the presence of endocrine disrupting pharmaceuticals (PhPs) in the aquatic environment may cause adverse effects, such as feminization in fish population^7^ and crocodiles^8^ and may also affect the behavior and migration movements of the salmons. Due to have low biodegradability, conventional treatment processes are not efficient enough for the removal of these compounds and lead to contamination in the receiving environment by bioaccumulation^9^. However, advanced oxidation processes (AOPs) are considered to be a promising methods for the solution of this problem.

AOPs are the oxidation methods based on the formation of reactive radical species (^·^OH-hydroxyl radical), which play an important role in the elimination of toxic organic compounds^10^. Advanced Oxidation Processes are used in wastewater treatment due to the formation of ^·^OH with high oxidation capacity. However, there are many variables affecting the processes; such as reaction time, pH, temperature, amount of catalyst and reagents^11^. Electrochemical processes, which place among the AOPs are based on production of hydroxyl radicals, which is the strongest oxidant type after the fluorine, and has at least as much reduction potential (E°: 2.8 V), that completely reacts selectively to organic contaminants by hydroxylation/dehydrogenation^12,13^.

Several anodic materials were used by the researchers for anodic oxidations, such as; PbO_2_, platinum (Pt), gold (Au), lead (Pb), SnO_2_, boron doped diamond (BDD) and carbon anodes. While some of the anode types selectively oxidize pollutants, those pollutants do not completely degrade (only the nature of the pollutant phase changes), the main disadvantage of other anode types is the rapid loss of effect due to surface contamination^14^. However, those anodes require high voltages to operate. The disadvantages of PbO_2_ anodes are that they have a shorter lifespan than other type of anodes^15^. Although BDD anodes have shown satisfying results for antibiotic removal, they have high capital cost and are not practical as Sn/Sb/Ni–Ti anodes^16^. In contrast to BDD anodes, Sn/Sb/Ni–Ti anodes are cost-effective. Additionally, 37% current efficiency could be reached at room temperature with Sn/Sb/Ni–Ti anodes^17^. Thus, Sb-doped SnO_2_ anodes are advantageous with their cost efficiencies and practical application. However, most of the other materials are not suitable due to their toxicity, instability and causing to the high costs. In contrast to these materials, even though Antimony (Sb) and Nickel (Ni) are known as toxic metals, because of the high stability and durability of Sn/Sb/Ni anodesachieve those problems sucessfully. Additionally, Sn/Sb/Ni anodes show very promising results in ozone production and electrochemical oxidation^18–22^. The most important advantage of these anodes in ozone production is their operation at relatively low voltage. On the other hand, they are likely to produce ozone in liquid and gaseous phases and do not need any input such as dry, moist air/pure oxygen and anodic gas source^21^. Also, the electrodes must be stable and remain stable at a voltage of 1.51 V (the ozone formation voltage) in electrochemical ozone generation with these anodes. Since the other anodes are not very stable, they are prevented by the formation of oxygen at a voltage of 1.23 V after a while, or a stability of 1.51 V can only be achieved at low temperatures^23^. Thus, in this study, it is investigated electrochemical oxidation of 50 mg L^−1^ cefoperazone, which place among the cephalosporin group of antibiotics, from aqueous solution with using novel Sn/Sb/Ni (Sb-doped SnO_2_-Ni) anodes. Goncalves et al. (2012) investigated catalytic ozonation of 50 mg L^−1^ sulfamethoxazole from aqueous solution^24^. Trovo et al. (2011)^25^ prepared simulated wastewater by adding amoxicillin antibiotic with a concentration of 50 mg L^−1^ for the Photo-Fenton process.

Cephalosporins have a great importance among the other antibiotic groups, because of having widespread usage in Turkey and worldwide (especially in northern european countries)^26^. These group of antibiotics are of the series of broad-spectrum semisynthetic antibiotics^27^. Among the cephalosporins, cefoperazone (CFP) is the third-generation type of semisynthetic antibiotic^28^. It is used against bacteria which are responsible for the infections of respiratory, urinary, skin, and female genital tracts having a broad spectrum of activity. First, cefoperazone was patented in 1974 and now, clinical use of cefoperazone in the U.S. is prohibited while, some of the European countries still use it (under the product name of Sulperazon)^29^.

However, most of the studies focuse on fluoroquinolone, trimethoprim, sulfonamide and macrolide antibiotics for the removal of them from water and wastewaters, while, just a little of them are made for cephalosporins and there are only a few studies about cefoperazone in literature. Furthermore, there is no such study on treatment of cefoperazone with these new generation Sb-doped SnO_2_-Ni anodes that make this study unique. Thus, this show that our study have a very important role in terms of filling this great gap in literature.

## Materials and methods

### Chemicals, anode preparation and reactor configuration

The simulated wastewater was prepared by adding sodium chloride (NaCl) and potassium chloride (KCl) (Merck, Darmstadt, Germany) as the electrolytes and cefoperazon antibiotic (from a local pharmacy, Bursa, Turkey) with a concentration of 50 mg L^−1^. Millipore Milli-Q ultrapure water (18 MΩ cm) was used for the preparation of simulated wastewater.

Cathodes were prepared in the size of 5 × 5 cm from platinized titanium material which was bought from NRK Electrochem Company, Cornwall, UK. Titanium (Ti) materials were purchased from Dexmet Company, USA (3Ti7-077FA) and then they were prepared by cutting the dimensions as 2.5 cm × 2.5 cm to prepare anodes. To remove all the impurities from Ti materials, they were dipped into the boiling oxalic acid solution (from Merck Company, Darmstadt, Germany). And then, theywere sonicated in ultrasonic bath for 10 min, three times. After drying process at room temperature, Ti materials were dipped into the pyrolysis solution having a molar rate: Sn/Sb/Ni: 500/8/1 for 2 min for dip coating process. And thus, the final product was obtained as a SnO_2_-based Ni/Sb anode material. SnCl_4_.5H_2_O (Tin (IV) Chloride Pentahydrate) and NiO (Nickel(II) oxide) were purchased from Alfa Aeser, MA, USA and Sb_2_O_3_ (Antimony(III) oxide) was bought from Merck Company, Darmstadt, Germany, which were used for dip coating process. The oven temperature for drying process was set at 105 °C for 15 min. Then, the oven temperature was set at 520 °C for 15 min time for the annealing process. This coating process was repeated for 19 times. However, the final process (20th) was performed for 75 min^30^. All of the chemical agents were at the purity of ≥ 99%.

**Figure 1:**
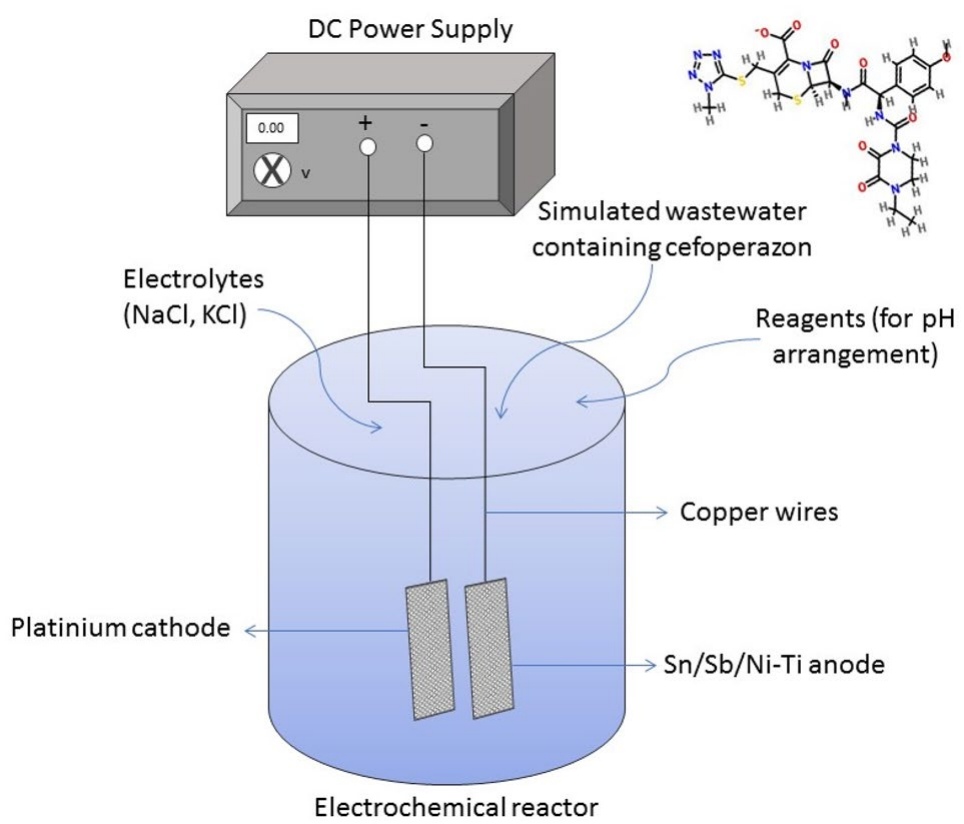
The electrochemical reactor configuration including Sn/Sb/Ni–Ti anode.

To design of reactor configuration for the electrochemical processes, 250 mL beakers were used. The electrodes (anode and cathode) were placed into the beakers mutually. The experiment conditions were 25 °C temperature and atmospheric pressure. The electrical current was created with DC power supply (Extech Instruments, 382280). In Fig. 1 it was stated the electrochemical reactor configuration including Sn/Sb/Ni–Ti anode.

### Analytical studies

A pH meter was used (Cyberscan 10) to measure of the pH values of the water samples. Chemical oxygen demand (COD) and total organic carbon (TOC) measurements were made according to the APHA Standard Methods (1995)^31^. TOC-L analyser (Shimadzu Company, Japan) was used for TOC analysis. Ultra performanced liquid chromatography (UPLC) with photodiode array (PDA) detector (Thermo scientific, US) was used to measure of CFP amount in water samples. The detector wavelengths were set at 254 and 270 nm. The column chosen for the UPLC measurements was C-18 type: 50 × 2.1 mm; 1.9 um (Hypersil Gold, Thermo scientific, US). The temperature of the column was 35 °C and the flow rate was 0.2 mL min^**−**1^. The mobile phase solvent was consisted of MeOH : H_2_O (0.1% formic asid): 40 : 60 (v:v). All of the measurements were repeated at least in triplicate. EDS (Energy Dispersive Spectroscopy) (EDAX, USA) and SEM (Scanning electron microscope) (Philips XL 30 SFEG, Netherlands), hpAFM (High Performance Atomic Force Microscope) (Nanomagnetic Instruments, Oxford, UK) and XRD (X-ray Diffraction) analysis were made for imaging and to perform qualitative and quantitative analyzes of the anode and to identify phases, crystallinity, and structures of the final products.

## Results

### The optimization of anode configuration

**Figure 2:**
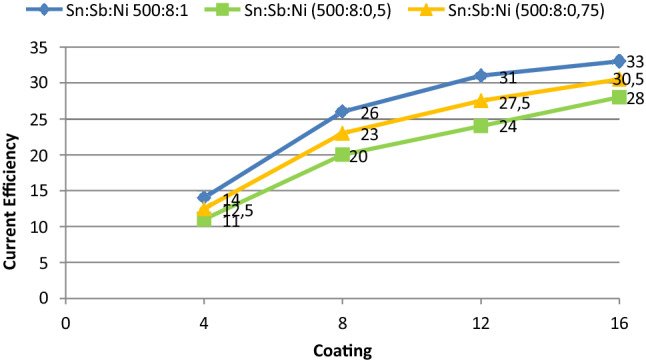
The effect of the number of coatings on the current efficiency in nickel-plated anodes.

The pyrolysis solution containing Sn:Sb:Ni was prepared in different molar ratios according to the prescription of the University of Hong Kong^32^. The effect of the number of coatings on the current efficiency was evaluated during electrolysis in 1 M HClO_4_^33^. It was observed that the current efficiency increases when the nickel ratio increases. At the same time, it is clearly seen that the current efficiency increases with the increase of number of coatings. Thus, it was decided to study with 500/8/1:S/Sb/Ni ratio. In Fig. 2 it is seen the effect of the number of coatings on the current efficiency in Nickel-plated (Sn/Sb/Ni) anodes.

### Determination of electrolyte type

**Figure 3:**
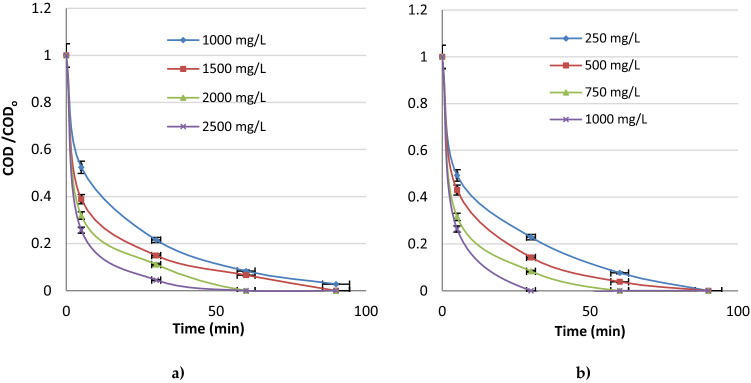
Effect of electrolyte type and concentration on chemical oxygen demand (COD) decrease with Sn:Sb:Ni: 500:8:1 anode (**a**) with NaCl and (**b**) with KCl addition (pH 8 and I: 50 mA cm^−2^).

At Fig. 3a, b it is seen the effect of electrolyte type on chemical oxygen demand (COD) removal from the water samples with Sn:Sb:Ni:500:8:1 anode. It was evaluated the effect of electrolyte type (NaCl and KCl) on electrochemical oxidation process with Sn:Sb:Ni:500:8:1 anode at various electrolyte concentrations. While the electrolyte type (NaCl, KCl) and concentration affected the removal efficiencies positively by increasing the conductivity and occuring chlorine gas and hypochlorid acid as efficient oxidants, addition of extra electrolyte may increase the process cost^34^.

The COD concentrations decreased to the zero after 60 min of anodic oxidation with 2000 mg L^**−**1^ NaCl addition, at 50 mA cm^**−**2^ current density and pH 8. However, COD concentrations decreased to the zero just after 60 min with 750 mg L^**−**1^ KCl.. Although high efficiencies were obtained in a short time for 2000 mg L^**−**1^ and 2500 mg L^**−**1^ NaCl conc., 750 mg L^**−**1^ KCl was regarded as the best electrolyte type and concentration due to the excess use of salt may increase the cost and chemical consumption. Yonar et al. (2019) investigated electrochemical color removal from organized industrial district (OID) wastewater treatment plants using new generation Sn/Sb/Ni–Ti:500/8/0.5 anodes and they studied with NaCl as the electrolyte at the range of 250–1000 mg/L. However, they found that, there was no need to addition of salt due to the study with real wastewater samples containing domestic wastewater^35^. Buyukada (2018) investigated TiO_2_ assisted photocatalytic ozonation of leather effluent and it was reported that increase in both of ozone and catalyst doses caused to increase in removals of chemical oxygen demand and turbidity^36^. Duan et al. (2020) reported electro-oxidation of ceftazidime antibiotic in real municipal wastewaters with PbO_2_-Ce and SnO_2_-Sb anodes. NaCl addition significantly enhanced the oxidation rate with 99% removal efficiency within 5 min of reaction with Ti/SnO_2_–Sb anode, while humic acid conc. inhibited electrochemical catalyzing^37^. Wang et al. (2022), added 20 mg/L NaCl, NaHCO_3_, Na_3_PO_4_, and HA (humic acid) as the electrolyte into the wastewater containing 4-chlorophenol (4-CP) antibiotic for the electrochemical oxidation of 4-CP by titanium suboxide anode with peroxymonosulfate. 4-CP removal efficiency was decreased at the rate of 97.4% and the corresponding kinetic rate was also decreased from 0.149 to 0.147 min^−1^ with the addition of Cl^−^ ions, which provide the best conditions^38^.

### Effect of potassium chloride concentration

**Figure 4:**
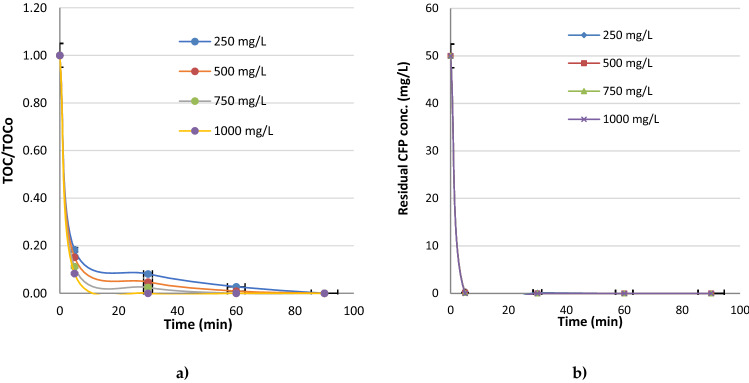
Effect of potassium chloride with Sn:Sb:Ni: 500:8:1 anode (**a**) TOC/TOC_o_ and (**b**) Residual CFP conc. (pH 8 and I: 50 mA cm^−2^).

At Fig. 4 it is shown the effect of potassium chloride on total organic carbon (TOC) and residual CFP removal with Sn:Sb:Ni: 500:8:1 anode. The effect of KCl (potassium chloride) concentration (mg L^−1^) on TOC and CFP were evaluated between 250 and 1000 mg L^−1^ KCl doses, at pH 8 and 50 mA cm^−2^ current density.

TOC was completely mineralized within 60 min reaction with 750 mg L^−1^ KCl as it was stated in a graph in Fig. 3. In the same way, residual CFP was decayed more efficiently just in 5 min with 750 mg L^−1^ KCl. Furthermore, after 30 min oxidation with 1000 mg L^−1^ KCl, the complete mineralization was achieved. However, the excess use of salt may increase the cost and chemical consumption. Thus, 750 mg L^−1^ was determined as the best dose for the electrochemical oxidation of cefoperazone with Sn:Sb:Ni: 500:8:1 anode. Sivrioğlu and Yonar (2016) investigated the removal of COD and color from textile wastewater with using Sn/Sb/Ni–Ti anode. Although the better efficiency was obtained at high NaCl concentrations, 1 g/L NaCl concentration was chosen as the best salt concentrationto prevent the high cost and environmental problems. Yao et al. (2019) investigated electrochemical oxidation of ammonia in dyeing wastewater with a Ti/PbO_2_ anode and Ti cathode and they obtained that, ammonia removal increased with increase in NaCl dose and thus, the removal efficiency reached to 100% with 1000 mg L^−1^ NaCl addition^39^. Hai et al. (2020) investigated the electrochemical oxidation of sulfamethoxazole (SMX) antibiotic with BDD (boron doped diamond) anode and complete removal of SMX and 65.6% COD removal, 40.1% current efficiency and 72 kWh kg/COD energy consumption rates were obtained with 0.1 M Na_2_SO_4_ after 3 h reaction at 30 mA/cm^2^ current density and pH 7^40^. Qian et al. (2019) investigated the effect of electrolyte addition on the electro-oxidation of tetracyclines (tetracycline, oxytetracycline, chlortetracycline) with Ti/SnO_2_-Sb_2_O_3_/PbO_2_ anode. While all the antibiotic compounds were completely removed with NH_3_·H_2_O-NH_4_Cl within 2 h, the removal efficiency was found below 80% with the addition of Na_2_HPO_4_-NaH_2_PO_4_, and after a 2 h of reaction time with Na_2_SO_4_, 82.4%, 83.6% and 88.4% removal rates were obtained for tetracycline, oxytetracycline and chlortetracycline, respectively^41^.

### Effect of pH

**Figure 5:**
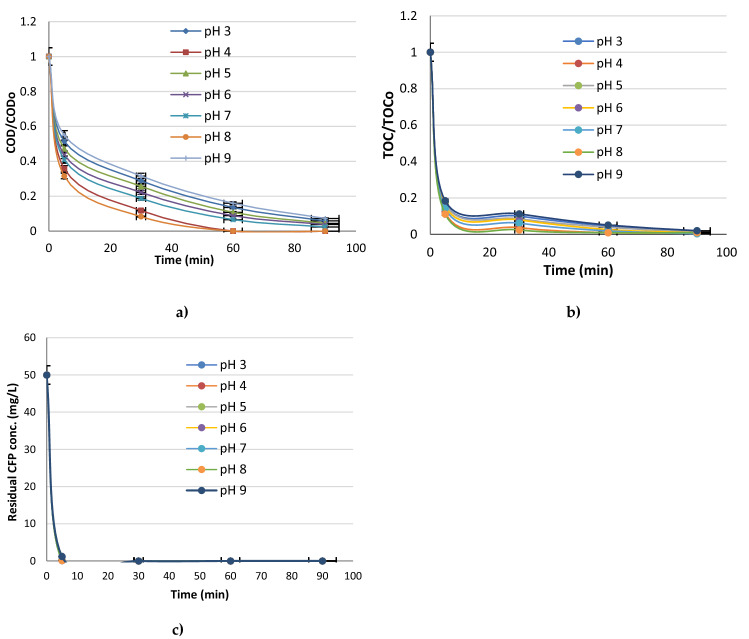
Effect of pH on (**a**) COD/COD_o_, (**b**) TOC/TOC_o_ and (**c**) Residual CFP conc. (KCl conc.: 750 mg/L, I: 50 mA/cm^2^).

At Fig. 5 it is shown the effect of pH on chemical oxygen demand (COD), total organic carbon (TOC) and residual cefoperazone (CFP) decay. At pH 8, the best removal efficiencies were obtained. Just after 60 min of reaction, COD and TOC was consumed completely, and the residual CFP was consumed just in 5 min at pH 8. Thus, according to the graphs in Fig. 4, pH 8 was found as the best pH value for the electrochemical oxidation of cefoperazone. According to these results, it was assumed that, it is possible to save costs by working at neutral pH value (pH 8) of water containing cefoperazone antibiotic, because the process doesn’t require extra labor and chemical costs to adjust the pH. Yonar et al. (2019) investigated electrochemical color removal from industrial wastewater with using new generation Sn/Sb/Ni–Ti: 500/8/0.5 anodes between pH 3 and 9. After 30 min of electrochemical reaction, COD and color removal efficiencies reached up to 98% and 99%, respectively at pH 8.2 (T: 25 °C) for colored industrial wastewater^35^. In a study made by Zakaria and Christensen (2014), it was reported that 100% removal efficiency was achieved within 5 min for Reactiv Blue 50 dye at a concentration of 1000 mg L^−1^ at pH 4.1, with using Sn/Sb/Ni anode and platinized titanium cathode fed to the membrane electrochemical electrode reactor system^42^. Abbasi, Soleymani, and Parsa (2015) performed an ozonation process on Rhodamine B molecules in aqueous solution using a reversible electrochemical ozone generator system with a titanium-based anode coated with nanocomposite Sn/Sb/Ni and they saw that the degradation efficiency could reach up to 99.5% at pH 3.7 for a 8 mg/L dye solution after 30 min^43^.

In the experimental studies of Sivrioğlu and Yonar^44^, COD and color removal was found as 98% and 99%, respectively as a result of electrochemical oxidation of dyeing wastewater at pH 3. However, although the natural pH value (pH 7.2) of the wastewater showed relatively lower efficiency (3%) compared to the acidic conditions, pH 7.2 was identified as the best in order to avoid extra pH adjustment step and chemical cost. At alkaline pH values, the potential of chlorine gas and hypochlorite ion formation could support the removal of organic compounds^45,46^. Yao et al. saw that, the increase of initial pH had a great effect on ammonia removal from dyeing wastewater with Ti/PbO_2_ anode and the best pH value was found as 8.3^39^. In a study of Kaur et al. (2018), with a Ti/RuO_2_ anode, removal of ofloxacin (OFL) occurred rapidly in the first 15 min of the reaction when the initial pH of the synthetic wastewater was between 2 and 9, and after 30 min, there was no any significant change. It was found that higher removal efficiencies were observed with lower pH values and after only 30 min of electro-oxidation 88.6% OFL degradation was observed at pH 2, while 68.6% OFL degradation was obtained within 30 min at pH 9^47^. Hai et al. (2020) investigated the removal of sulfamethoxazole (SMX) with boron-doped diamond anode at pH 3, pH 7 and pH 11 at a current density of 30 mA cm^−2^. It was observed that higher removal efficiencies were obtained at neutral pH (pH 7) compared to those at pH 3 and pH 11^40^.

### Effect of current density

**Figure 6:**
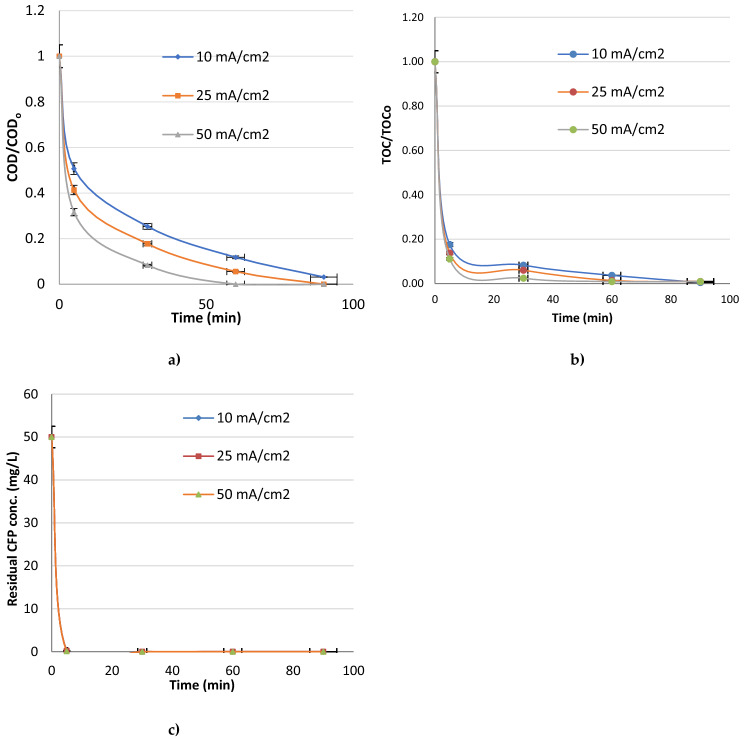
Effect of current density on (**a**) COD/COD_o_, (**b**) TOC/TOC_o_ and (**c**) Residual CFP conc. (KCl conc.: 750 mg/L, pH 8).

The current density has an active role in reaction kinetics and thus, affects the electrochemical reactions significantly^46^. The best current density value was found as 50 mA cm^−2^ due to obtainhigher removal efficiencies in shorter times. In the aqueous solutions, active oxidants occur increasingly at higher current density values thus, the removal efficiencies increased generally with the increase of current density in this study. Yonar et al. (2019) investigated electrochemical color removal from industrial wastewater using new generation Sn/Sb/Ni–Ti: 500/8/0.5 anodes, between 10 and 100 mA/cm^2^ current density values. The best current density value was found as 50 mA cm^−2^ with 4.05 kWh gCOD^−1^ energy consumption and the current density was observed as the most effective parameter for COD and color removal^35^. Duan et al. (2020) reported electro-oxidation of ceftazidime antibiotic in real municipal wastewaters with PbO_2_-Ce and SnO_2_-Sb anodes. While 99.37% of ceftazidime degradation and 95.52% COD removal was achieved with Ti/SnO_2_–Sb anode, 75.15% ceftazidime degradation and 83.54% COD removal was obtained with Ti/PbO_2_–Ce anode, under 4 mA cm^−1^ current. Wen et al. (2019) investigated mineralization of cefoperazone (50–300 mg L^−1^) in acidic medium with photoelectro-Fenton with microwave discharge electrodeless lamp irradiated by using RuO_2_/Ti and boron doped diamond (BDD) anode. BDD microwave discharge electrodeless lamp photoelectro-Fenton gave 88% mineralization under the best conditions of 0.36 A^48^. Wang et al. (2022) studied electrochemical oxidation of 4-chlorophenol (4-CP) by titanium suboxide anode with peroxymonosulfate and they saw that the degradation efficiency was obviously increased with the increase of current density from 1 to 10 mA/cm^2^^38^. Zeng et al. (2022) investigated electrochemical oxidation of sulfamethoxazole (SMX) in natural water and wastewater with TiO_2_ nanotube array based electrocatalytic membrane. The removal rate of SMX was 86.1% and the energy consumption was 0.55 kWh/m^3^ with one dimensional nanostructure and the degradation efficiency of SMX was strongly affected by the current density. Both hydroxyl radicals and direct electron transfer affected the sulfamethoxazole degradation, while the sulfate radicals could be ignored^49^. Yao et al. (2019) reported that ammonia removal increased significantly with increase of applied current density from dyeing wastewater with Ti/PbO_2_ anode and Ti cathode and the best current density value was found as 20 mA cm^−2^^39^. Hai et al. (2020) investigated the removal of sulfamethoxazole with BDD anode and according to the results of the study, SMX decomposed completely after 1 h of electrochemical reaction at a current density of 45 mA/cm^2^, while it decomposed after 3 h at the current density values of 15 mA/cm^2^ and 30 mA/cm^2^^40^. Qian et al. (2019) investigated the effect of the current density parameter on the electro-oxidation of tetracyclines with SnO_2_-Sb_2_O_3_ and PbO_2_ doped Ti anodes. They reported that the degradation of antibiotics within a reaction of 2 h without power supply was less than 2% indicating that the adsorption of antibiotics on the anode surface could be negligibly low. With the increase of current density, the removal rates of tetracyclines gradually increased and the removal rates were 98.1%, 97.6% and 99.5% for tetracycline (TC), oxytetracycline (OTC) and chlortetracycline (CTC), respectively at a current density of 15 mA/cm^2^ for 2 h. However, the removal rates of tetracycline, oxytetracycline and chlortetracycline were found to be 79.5%, 82.0% and 90.3%, respectively, at a current density of 5 mA/cm^2^^41^. In Fig. 6 it is shown the effect of current density (10–50 mA cm^−2^) on total organic carbon (TOC) and residual CFP removal with Sn:Sb:Ni: 500:8:1 anode (pH 8 and KCl conc.: 750 mg L^−1^).

### Evaluation of total intermediate product formation

**Figure 7:**
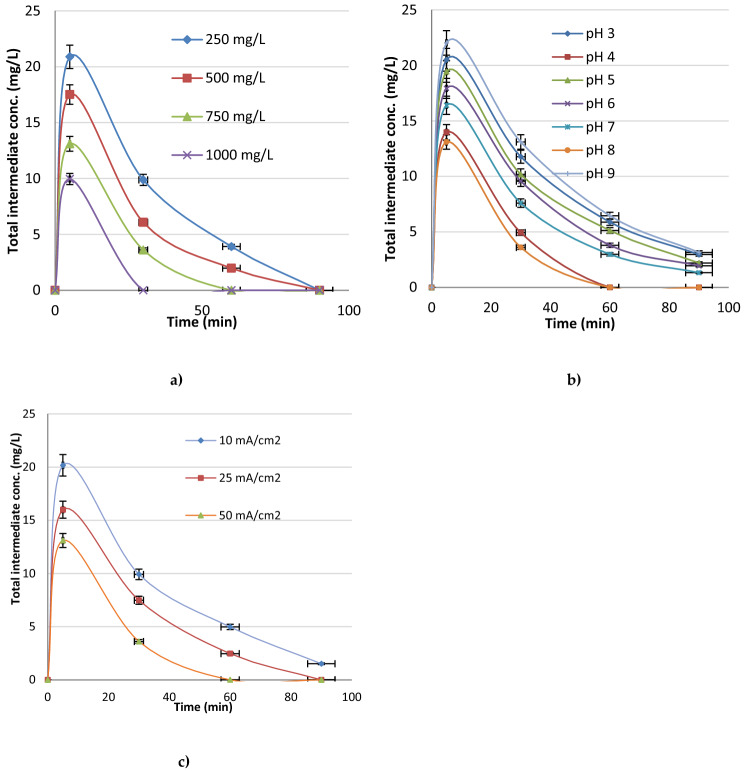
Total intermediate product formation (**a**) effect of KCl concentration (pH 8 and I: 50 mA cm^*−*2^) (**b**) effect of pH (KCl conc.: 750 mg/L, I: 50 mA/cm^2^) (**c**) effect of current density (KCl conc.: 750 mg/L, pH 8).

Total intermediate product formations are shown in Fig. 7 according to the effect of KCl concentration, pH and current density.According to the graphs in Fig. 7, much lower intermediate products were occurred with KCl addition and the organic content was almost completely mineralized just in 60 min with 750 mg L^*−*1^ KCl. The lowest occurances of total intermediate products were obtained at pH 4 and pH 8 with the current density value of 50 mA/cm^2^.

Yahya et al. (2016) investigated the ability of Electro-Fenton process with carbon-felt cathode and Pt anode for degradation and mineralization of levofloxacin (LEV) in aqueous solution. 400 mA current value was observed to be optimum. Chemical oxygen demand and mineralization degree reached to > 91% at the end of 6 h of reaction. A number of intermediate products were identified by using HPLC and LC–MS. N atoms in LEV were released as NH^+4^ and NO^−3^ ions. Nitrogen atoms mainly transformed into NH^+4^ rather than NO^−3^. The concentration of NH^+4^ reached to 0.28 mM after 300 min, while that of NO^−3^ reached to the zero after 300 min. The nitrogen loss could be explained by the formation of volatile nitrogen compounds and the presence of oxamic acid that is hardly oxidizable by hydroxil radicals^50^. The biggest advantage of the electrochemical oxidation processes is that, pollutants are completely oxidized ideally. However, it is known that, organic compounds with high permanence are more concentrated with phase change instead of oxidation completely in classical processes.Thus, electrochemical oxidation processes are highly promising^26^.

### SEM–EDS, XRD and HpAFM analyzes

**Figure 8:**
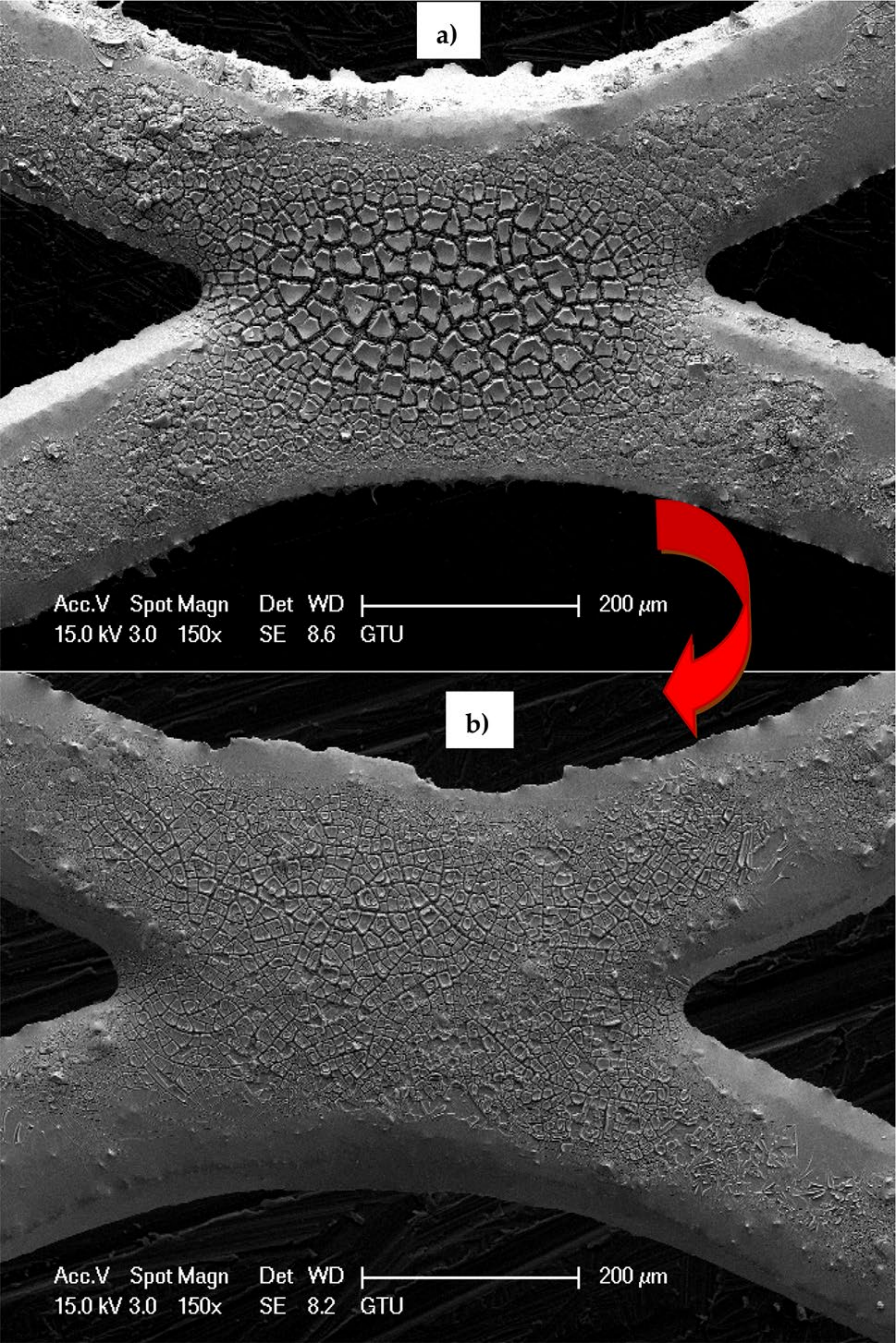
(**a**) Typical SEM images (× 150, bar = 200 μm) of Sn/Sb/Ni: 500/8/1 anodes. (**a**) Unused anode (clean product), (**b**) used anode (contaminated product).

To image and to perform qualitative and quantitative analyzes of the anode as a final product and to identify phases, crystallinity, and structures of it SEM–EDS, hpAFM and XRD analyzes were made out. In Fig. 8a, b it is seen typical SEM images (× 150, bar = 200 μm) of Sn/Sb/Ni: 500/8/1 anodes (clean and used anodes, respectively). The developments in nanocomposite material applications in engineering (mechanical, optical, electrical and magnetic applications) are promising^51^. Thus, electrochemical oxidation processes with nanocomposites are promising with their easy applicability and low energy requirements^52,53^.

**Figure 9:**
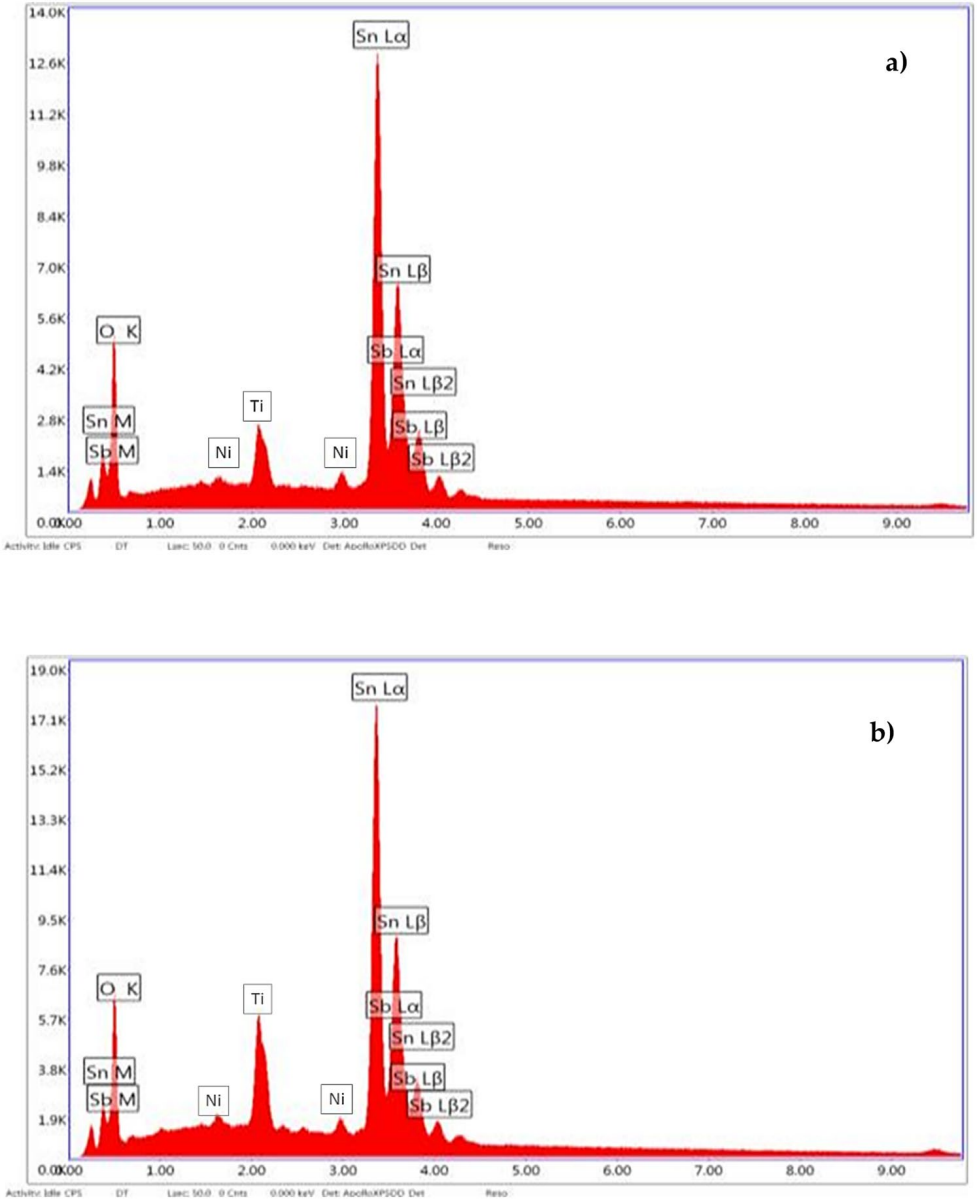
EDS spectra of the anodes (**a**) unused anode (clean product), (**b**) used anode (contaminated product).

The weight and atomic percentages and the peak intensities in Fig. 9a, b were given in Table 1 for the anode characterization. The peaks of the elements (Sn, Ni, Sb and Ti) were identified by the analysis of anode material. Coating on the anodes (Ni/Sb-SnO_2_) was thin to enough detection of Ti underlying. Typical SEM micrographs of the anode intersections (assuming thicker coating than strands)^30^ at × 150 magnification are shown in Fig. 8 for used (contaminated) and unused (clean) anode, respectively. It was observed that the anode materials showed a cracked morphology because of the coating process for clean anodes as stated in other studies^54^ that was occurred by thermal shocking which is seen generally while cooling suddenly after taking of the anodes from oven. Cracked morphology is seen in thicker anodes mostly with large splits having three dimensional (3D) view^55^. However in contaminated (used) anodes, a smoother surface was observed that resulting from the coating of surface area with ions (carbon) and salts (Fe_3_(PO_4_)_2_(OH)_2_) passing from the solution.

**Table 1:** Weight and atomic percentages and the peak intensities in EDS spectra of the anodes.

	Element	Weight %	Atomic %	Net peak int	Net peak int. error
Clean anode	Sn	81.82	49.83	2683.22	0
	Sb	8.15	4.84	239.81	0.06
	Ni	6.02	27.20	276.68	0.006
	Ti	25.075	7.52	345.855	0.008
Contaminated anode	Sn	82.65	52.1	3270.5	0
	Sb	8.18	5.03	290.4	0.06
	Ni	1.84	8.58	100.97	0.002
	Ti	22.925	107.2	1262.15	0.02

Christensen et al. (2013) investigated the effect of Ni and Sb oxide precursors and composition of the anode in ozone production with 1.0 M HClO_4_^30^. Typical SEM images of the anodes (93.3, 6.0 and 0.7% Sn, Sb and Ni respectively) which were taken from the intersection (× 5000 magnification) showed cracked morphology (sources from thermal shock during the cooling suddenly after withdrawing of anodes from oven), while the coating on strands showed a smoother morphology assuming have thinner coating than the intersection. With EDS spectra of the anode it was seen that, the peak with 4.52 eV may be sourced from Ti underlying that could be derived from thinner catalyst coating than the strands. Christensen et al. (2012) studied the effect of Ni/Sb-SnO_2_ loading on electrocatalyst and they observed with the SEM images that the electrode was thicker and had very little pores having typical “cracked morphology” with deep crevices^55^. Moreover, it was observed that Ni/Sb-SnO_2_ coating was thin sufficiently to able to detect Ti element underlying. Zhi et al. (2017) investigated the degradation of tetracycline antibiotics with Ti/SnO_2_-Sb anodes and used the sol–gel technique to coat the anode^56^. Although there were some cracks ranging in size from 1 to 10 μm, SEM images revealed that the anode surface was generally solid and smooth. In addition, they concluded that the formation of these cracks could result in gradual inhibition of the anode during the electrochemical process. Qian et al. (2019) reported that the Ti substrate surface pretreated with Ti/SnO_2_-Sb_2_O_3_/PbO_2_ anode is irregular and crusted, which is presumed to be formed as a result of oxalic acid application^41^. Thus, they stated that it may be beneficial to add SnO_2_-Sb_2_O_3_ and PbO_2_ as interlayers and active layers, respectively.

**Figure 10:**
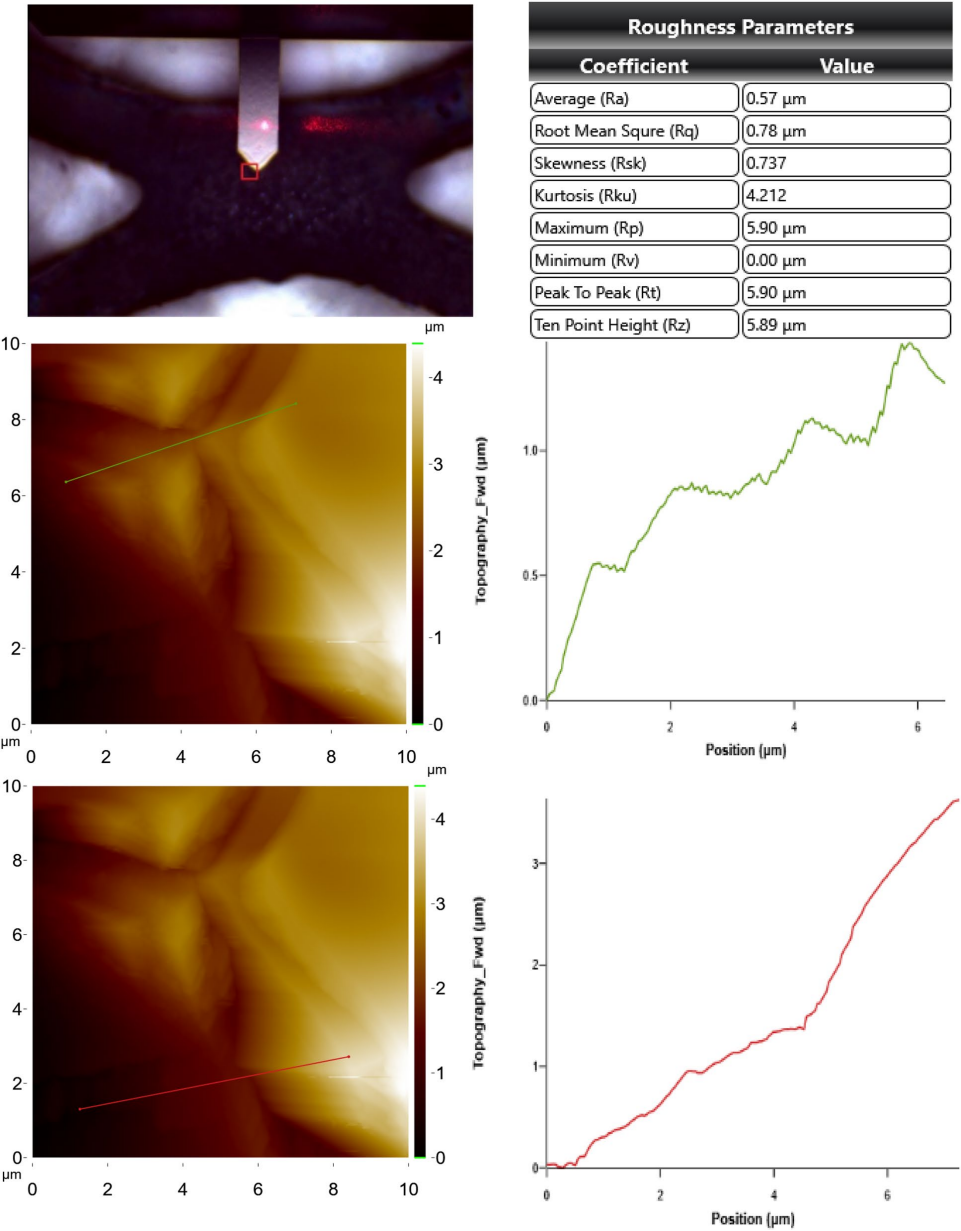
AFM images for upper cross section and bottom cross section of the clean anode.

**Figure 11:**
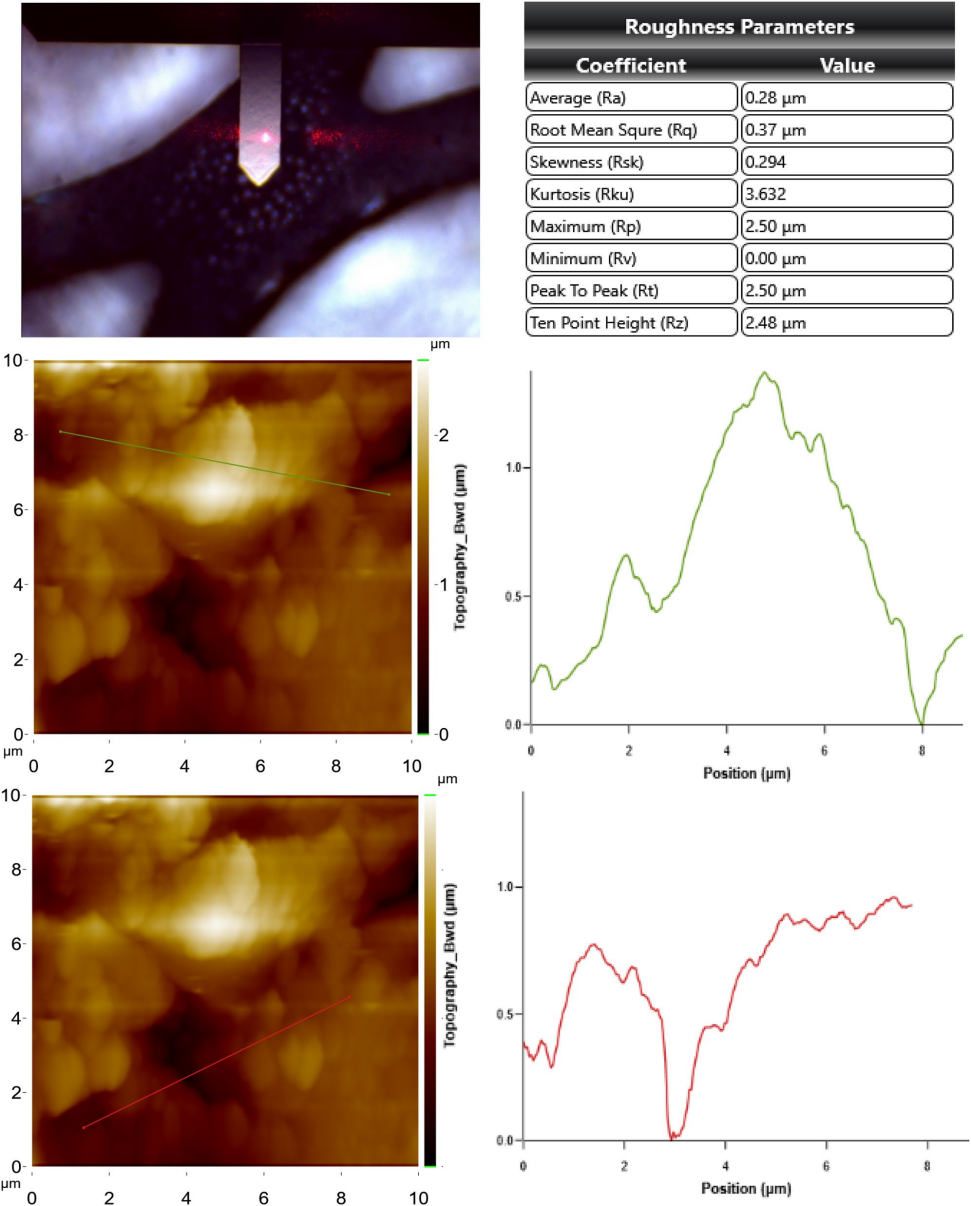
AFM images for upper cross section and bottom cross section of the contaminated anode.

**Figure 12:**
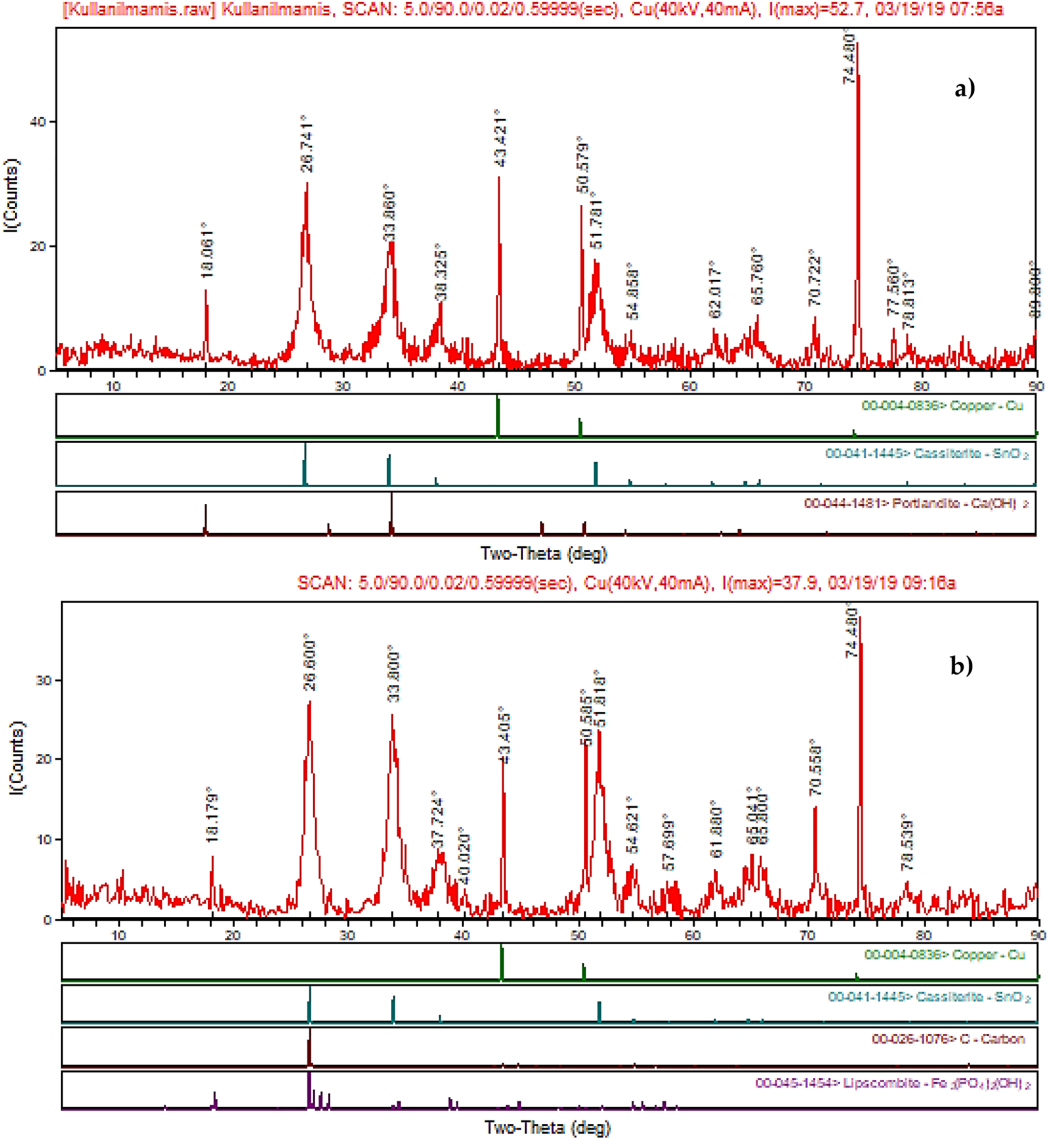
XRD analysis of the anodes (**a**) clean anode and (**b**) contaminated anode.

In addition, Figs. 10 and 11 show AFM images of the anodes showing the topographical height change during the electrochemical oxidation process. It has been observed that there is a very high difference between contaminated and clean anode in terms of topographic height. While topographic height differences tend to increase in parallel for upper cross and bottom cross section for the clean anode; irregularities were observed for contaminated anode, which is thought to be due to ion transfer from the aqueous solution. However, it is known that the physicochemical properties of the anodes are directly related to the preparation methods. Composition ratios, particle size, surface structure, specific surface area and bonding force directly affect the performance of the anode^57^. Figure 12 shows the XRD results of clean and contaminated Sn/Sb/Ni–Ti anodes. Consequently, XRD results confirmed other SEM–EDS findings. It was observed that the surface of the used (contaminated) anode was filled with other ions (carbon) and salts (Fe_3_(PO_4_)_2_(OH)_2_) may source from the solution. It was worked a total of 300 h with the anodes, and thus, it was obtained that the anode material is not corroded significantly.

## Conclusions

In this study, electrochemical oxidation of aqueous solution containing antibiotics with using new generation Sn/Sb/Ni: 500/8/1 anodes were carried out. pH, electrolyte type and conc. and current density parameter effects and total intermediate product formation was evaluated.

KCl was found as the best electrolyte type affecting the elctrochemical reactions positively the most even in lower concenrations. Thus, according to the results of the study, it could be possible to obtain higher removal efficiencies with real water/wastewater samples (assuming include KCl ions, mostly) without additon extra chemicals as the electrolyte. pH 8 is the neutral value of the aqueous solution containing antibiotics was obtained as the best due to provide higher removal efficiencies. Therefore, it could be possible to operate process easier and more economically by working at neutral pH values, due to there is no need to additional chemical cost for extra pH arrangement step. The removal efficiencies increased generally with the increase of current density, because of the occuring of active oxidants increasingly at higher values. 50 mA/cm^2^ was found as the best for current density, having full mineralization after 60 min. In our study, it was obtained more efficient results at lower current densities (50 mA/cm^2^) with Sn/Sb/Ni anode, compared to the most of the researches on electrochemical treatment of antibiotics. According to the results of the study, it is thought that the electrochemical oxidation processes could be carried out in real wastewaters without need to adding extra salt and pH arrangement step. So, it is easy and economical way to perform electrochemical treatment processes with Sn/Sb/Ni–Ti anodes with very high removal percentages for wastewaters containing antibiotics. Also, it needs less reaction time than the conventional treatment methods. At this respect, working with these anodes is promising for the future studies. In contrast to the other materials used for anode production, Sb-doped SnO_2_-Ni anodes don’t have much more toxicity and instability causing to the high costs. Additionally, these new generation anodes show very promising results in ozone production. However, most of the studies have focused on fluoroquinolone, trimethoprim, sulfonamide and macrolide for the removal of them from aquatic environments, while, just a little of them have been made for cefoperazone antibiotic. There are just only a few studies made about cefoperazone. The fact that there is no such studies ontreatment of these antibiotics with these new generation anodes has made this study unique.

## Supplementary Information

Supplementary Information 1.

Supplementary Information 2.

## Data Availability

All data generated or analysed during this study are included in this published article [and its supplementary information files].
